# Stroke survivors’ preferences regarding study participation in rehabilitation research

**DOI:** 10.1186/s12874-022-01521-z

**Published:** 2022-01-30

**Authors:** Emma Carlstedt, Eva Månsson Lexell, Agneta Ståhl, Arne Lindgren, Susanne Iwarsson

**Affiliations:** 1 Department of Health Sciences, Lund University, Box 157, 221 00 Lund, Sweden; 2 Department of Neurology, Rehabilitation Medicine, Memory Clinic and Geriatrics, Skåne University Hospital, Lund-Malmö, Sweden; 3 Department of Technology and Society, Lund University, Lund, Sweden; 4 Department of Clinical Sciences Lund, Neurology, Lund University, Lund, Sweden

**Keywords:** Participants’ recruitment, Recruitment facilitators, Rehabilitation, Survey administration modes; users’ perspective

## Abstract

**Background:**

To pursue high quality research, successful participant recruitment is essential, but recruitment rates are often low. This is specifically true in target populations with impairments, for instance, among stroke survivors. Previous studies focusing on recruitment have mainly relied on information from professionals, and there is therefore a need to contribute with new methodological insights to how potential rehabilitation research participants describe their interest and preferences to participate in research. The purpose of this study was to generate knowledge about stroke survivors’ interest in participating in rehabilitation research, reasons for being interested or not, and preferred forms and foci of rehabilitation interventions. An additional aim was to describe preferences regarding survey administration modes and processes for recruitment to studies.

**Method:**

This cross-sectional study recruited Swedish residents who had sustained a stroke, initially by using advertisement on the National Stroke Association’s website, flyers posted at local occupational and physical therapy offices and at local stroke/senior organization meetings. Secondly, participants were recruited through a local stroke register. The survey, administered either in a paper form returned by postal mail; online or as a phone interview with 128 stroke survivors.

**Results:**

Most of the participants were interested in participating in rehabilitation research, particularly younger persons (*p* = 0.001) and those closer to stroke onset (*p* = 0.047). Contribution to research, possibility to try new rehabilitation interventions and meeting others in the same situation were reasons that attracted an interest to participate. Other important aspects were related to motivation, individual needs, as well as how skilled the people who provided the intervention were. Participants preferred group-based programs, and programs focusing on regaining lost functions were highly requested. A majority wanted to be contacted through postal mail (70%) and most of them (90%) used the paper form to respond to the survey.

**Conclusions:**

A range of personal and external aspects, including challenges related to digitized administration modes, should be considered to achieve high participation rates in rehabilitation research targeting stroke survivors. The importance of addressing individual needs and prerequisites in an individualized manner should not be underestimated and might be a useful strategy to recruitment success.

## Background

To pursue high quality research, successful participant recruitment is essential but response rates are often low in health-related surveys [1] and randomized controlled trials [2]. This paper aspires to contribute with new knowledge with the potential to nurture methodological awareness and strategies related to recruitment of study participants.

Recruitment to rehabilitation research is particularly exposed to risk of selection bias as the target populations are people with impairments [3], for example, people who had a stroke. Many stroke survivors need rehabilitation long-term after onset [4]. In Sweden, the societal costs for stroke have been increasing, especially the direct costs related to residential care and home assistance [5]. Thus, beyond individual wishes to recover and adapt to an altered life situation post stroke, there are economic incentives to provide cost-efficient rehabilitation alternatives. There is therefore a need to develop evidence-based and efficient rehabilitation programs that meet individual and societal needs. Thus, effective strategies to increase recruitment to studies within rehabilitation research are therefore needed [6].

Challenges to study participant recruitment are related to individual and societal expectations, for example, stroke survivors’ lack of interest and transport possibilities, health issues [7, 8], old age [8] and inability to give informed consent [8, 9]. Challenges can also be related to those who provide the intervention, and the requirements for their work, and to the study as such. Simple entry criteria and study procedures are of importance [9], and diverse ways to recruit in different settings [10] have also been suggested. Still, previous studies have mainly relied on information from professionals [8, 9, 11], and there is a need to directly ask potential participants about their interest in, and preferences related to participate in rehabilitation research.

The purpose of this study was to generate knowledge about stroke survivors’ interest in participating in rehabilitation research, reasons for being interested or not, and preferred forms and foci of rehabilitation interventions. An additional aim was to describe preferences regarding survey administration modes and processes for recruitment to studies.

## Methods

### Design

We designed a cross-sectional study with data collection based on a self-administered survey, created and piloted for the purposes of the present study, utilizing different recruitment methods.

### Description of participant recruitment

We recruited people who had sustained at least one stroke event in adulthood (≥ 18 years), were resident in Sweden and able to independently or through support from another person fill in a self-administered survey in Swedish. Multiple recruitment modes were used. As a first round of recruitment, we used the two following methods; i) advertisement on the National Stroke Association’s website, and ii) flyers posted at local occupational and physical therapy offices, at local stroke organization member meetings and senior organization meetings. Due to the considerable difficulties experienced while using the two modes, direct postal surveys were sent to the stroke survivors on the local Lund Stroke Register (LSR) [12] to facilitate recruitment in a second round. Because recruitment through the patient register mode was made in a second round, subjects in this sub-sample were asked to state if they had already responded to the present study as part of the earlier phase of recruitment. If the answer was positive, they were asked to return the survey blank. The recruitment to the study went on during 3 months and offered three administration modes; 1) paper form, returned by postal mail, 2) online, using a web address, or 3) phone interview by one of the authors (EC).

### Survey questionnaires

A self-administered two-part questionnaire constituting the survey was constructed in an interactive process by the co-authors who had different professional/disciplinary backgrounds and expertise. The first part was study-specific and included 18 questions. In addition to demographic questions (e.g., sex, age, education, living conditions, time since stroke), the questionnaire captured interest in rehabilitation interventions (one question with a 0–10 scale; 0 = not at all interested; 10 = very interested) and in participation in rehabilitation research, reasons for the interest, contact mode preferences, as well as preferred intervention forms and foci. The latter questions had a multiple-choice structure, with the possibility for more than one response option. In addition, the open-ended question “What do you think is important for researchers to consider when recruiting stroke survivors’ to rehabilitation research?” was included. Prior to the data collection, the questionnaire was piloted with five stroke survivors (not included in the study sample). Only minor wordings were altered after the discussion.

The second part included questions from three established questionnaires: The Stroke Impact Scale, 2.0 (SIS) [13]; the General self-efficacy scale (GSE) [14, 15], and the Geriatric Depression Scale (GDS-20) [16]. We included four physical items and eight cognitive items from the SIS, measured by a 5-point scale (1 = no strength at all/ extremely difficult to 5 = a lot of strength/not difficult at all). Higher scores indicate less impact on strength/cognition. Mean scores of the items within each domain were calculated and transformed into percentage values [13]. We included all ten items from the GSE [14, 15], rated on a four-graded scale (1 = Not at all true; 4 = Exactly true). Higher score indicated higher sense of general self-efficacy. The GSE scale is valid [17] and reliable in stroke samples [18]. The full questionnaire of the GDS-20 [16] were used. It comprises 20 items on a dichotomous scale (yes/no); > 5 points indicate a possible depression. The GDS-20 has been used frequently among participants in different ages, with stroke or other diagnoses [19, 20]. In Table 1, an overview of the different recruitment modes, information channels and survey distribution is presented.

**Table 1:** Overview of the recruitment modes, information channels and survey distribution

Recruitment Mode	Information channel	Survey distribution
*Advertisement*	- National Stroke Association’s website, Facebook account and journal for members - Flyers posted at local OT/PT^*^ offices in the south of Sweden (*n* = 3)	Those interested answered by phone or e-mail that they wanted to receive the survey (*n* = 38)
*Presentations*	- One of the authors (EC) presented the study at local stroke organization member meetings (n = 5) in the south of Sweden - One of the authors (SI) presented the study at senior organization meetings (*n* = 5) in the south of Sweden	Surveys distributed in person during the presentations (*n* = 94)
*Patient register*	- Lund Stroke Register (LSR)^†^ (persons with stroke onset Mar. 2013-Feb. 2014)	Surveys by postal mail (*n* = 200)

^*^OT/PT Occupational therapists/Physiotherapists ^†^A hospital-based stroke register at Skåne University hospital in Lund, Sweden, covering eight municipalities [11, 12]

### Data analysis

The numerical data from the survey were analyzed using descriptive statistics, and the qualitative data from the open-ended question were analyzed through thematic analysis [21].

To improve the interpretability of the results the “interested in participating in rehabilitation research” variable was dichotomized. Comparisons between interested/not-interested participants were computed to identify differences in relation to age, gender, time since stroke, SIS domains, GSE and GDS. Mann-Whitney U or Chi-square tests were applied as appropriate. Missing values were imputed using documented rules for SIS, GSE [13, 22] and GDS-15 (no documentation was found for GDS-20) [23]. For comparison between response modes and administration modes, respectively, Chi-square or Kruskal-Wallis tests were used. When applicable, pairwise Mann-Whitney U test with Bonferroni correction was utilized. Statistical significance was set at *p* < 0.05. The SSPS Statistics 22.0 software (IBM Corporation, Armonk, New York, USA) was used to analyze the data.

## Results

### Participants’ characteristics

A total of 128 participants (52 women and 41 men) with a median age of 73 years were included in the study. The median time since stroke onset was 1 year or more for over 90% of the participants. Most of them lived with a partner (66%), in a single-family house (54%), and in an urban area (86%). Participant characteristics are further described in Table 2.

**Table 2:** Participant characteristics, *N* = 128

Variable	*N* = 128^*^
Gender, n (%)	
Female	52 (41)
Age, years	
Median (min-max)	73 (34–102)
Time since most recent stroke, n (%)	
0–6 month	3 (3)
> 6 month, but <one year	4 (3)
> one year, but <five years	87 (70)
≥ five years	30 (24)
Living condition, n (%)	
Alone	43 (35)
Partner	66 (54)
Partner and children	9 (7)
Children	5 (4)
Housing condition, n (%)	
Single-family house	65 (54)
Multi-family house	53 (44)
Other	3 (3)
Geographical area, n (%)	
Urban (highly and semi urban towns)	109 (86)
Highest education, n (%)^†^	
Primary school	21 (17)
Secondary school	52 (42)
University	52 (42)

^*^Missing N = 1–7. ^†^Due to rounding of decimals, the total sum exceeds 100%

### Response rates and preferred administration modes

In total, 332 surveys were distributed, resulting in a 39% response rate (*N* = 128). The data collection were performed prior to the covid-pandemic. Among the 128 people who answered the survey, the response rates for each recruitment mode were 90% (advertisement), 37% (flyers/presentations at meetings) and 30% (patient register). There were no significant differences in response mode across gender, but age differed significantly between the advertisement and patient register modes (*p* < 0.001; higher age in the patient register sub-sample). In addition to the 128 responses, 10 questionnaires were resent blank with a note, or a family member phoned to explain why no response was made (e.g., health issues). The vast majority delivered their completed survey in paper form (90%). There were a few online (9%) and phone (1%) responses. No differences in administration mode were observed across gender (*p* = 0.906), age (*p* = 0.062) or recruitment mode (*p* = 0.110).

### Interest in participating in rehabilitation interventions

Overall, the participants reported a high interest to participate in rehabilitation interventions. A median of 8 was reported for ‘regaining lost physical and cognitive functions’ as well as for ‘learning strategies to manage tasks and situations that can be difficult after stroke’, while ‘finding new/alternative ways of performing daily activities (including provision of assistive devices/housing adaptations)’ had a median of 7.

### Interest in participating in rehabilitation research

A majority of the participants (*n* = 105; 82%) indicated that they were interested in participating in rehabilitation research. More than 50% (*n* = 69) replied “definitely interested” and about a third “maybe” interested. The remaining (less than a fifth) answered “definitely not interested”. Younger participants were more interested than older (*p* = 0.001) and there was a lower interest among those who had their stroke onset a longer time ago, compared to those who recently had their stroke (*p* = 0.047). There were no statistically significant differences between those interested in participating in rehabilitation research and those that were not across each of the participants’ gender, strength, cognitive impact, general self-efficacy or possible depression, see Table 3.

**Table 3:** Comparisons between participants interested and not interested in RR^*†^, *N* = 128

Variable	Interested *n* = 102 (82%)	Not interested *n* = 23 (18%)	*p*-value^**^
Gender, n (%)			
Female	44 (43)	7 (30)	*0.263*
Age, years			
Median (min-max)	72 (34–91)	79 (63–102)	*0.001*
Time since most recent stroke, n (%)^‡^			*0.047*
0–6 month	3 (3)	–	
> 6 month, but <one year	3 (3)	1 (4)	
> one year, but <five years	64 (65)	21 (91)	
≥ five years	28 (29)	1 (4)	
SIS, strength^§^			*0.334*
Median (min-max)	63 (0–100)	69 (25–100)	
SIS, cognition^§^			*0.708*
Median (min-max)	88 (9–100)	89 (25–100)	
GSE^| |^			*0.263*
Median (min-max)	31 (10–40)	31 (12–40)	
GDS^#^			
Possible depression >5p, n (%)	60 (61.9)	12 (63.2)	*0.915*

^*^Missing, *n* = 3. ^†^We did not calculate for any ethnical differences. ^**^X^2^ and Mann-Whitney U test

^‡^Due to rounding of decimals, the total sum is lower than 100%. ^§^SIS=Stroke Impact Scale (the higher proportion, the lower impact on strength/cognitive difficulties). ^| |^GSE = General Self-Efficacy Scale (=higher score indicate higher sense of general self-efficacy). ^#^GDS = Geriatric Depression Scale

### Reasons for participants’ interest in rehabilitation research

The three most reported reasons for participants’ interest in rehabilitation research were to contribute to research (69%), try new rehabilitation programs (48%), and meet others in the same situation (46%) (Table 4). The most frequent reason for not being interested was revealed as no perceived need for rehabilitation (18%). Further, difficulties to travel to and from the rehabilitation setting (11%), insufficient energy to participate (9%), uncertainty of being able to manage (8%), not interested to participate in research at all (8%), severe health issues (6%) and no time to participate (4%) were indicated as other barriers.

**Table 4:** Reasons for being interested in participating in RR, *N* = 128^*†^

Reason (item)	%
Contribute to research	69
Possibility to try new rehabilitation interventions	48
Meet others in the same situation	46
Possibility to take a break if I get tired	32
Get help to get to and from the setting, if the program is performed outside my home	22
Dissatisfaction with rehabilitation that I have received so far	19
Compensation for travel expenses and lost income	18
Investigators use an easy language	18
Possibility to bring a relative	16
Nothing, since I am not interested to participate in RR	11
Know the investigators from before	5

^*^Missing, *n* = 2. ^†^It was possible to choose several response options

### Preferences for participating in rehabilitation research

If invited to participate in rehabilitation research, the most preferred information channels were through postal mail (70%), personal meetings (36%) or phone calls (34%). Advertisement through the Internet (e.g., patient organization sites, social media) was less attractive (21%), and so were newspaper (3%) and TV/radio (3%). Sixty-three (49%) responded to the open-ended questions, and indicated that information initially should be directed to family members, and that programs should be conducted not long after the stroke event: *“Long time since the stroke limits the interest”*. Contrasting this, others stated that rehabilitation was important also many years after stroke:” *Recruit also persons who had their stroke onset a long time ago (20–30 years)! Improvements can occur even many years after the stroke”.*

More than two thirds (67%) expressed a high interest for participating in group-based interventions conducted in, for example, primary care, hospital or university facilities. This was also reflected in the responses to the open-ended questions. Almost one-third (29%) were interested in interventions where the people who provided the intervention made home visits, and 19% in interventions delivered by phone. Interventions combining home visits and phone calls attracted 13%, Internet/video based interventions 14% if performed individually, and 3% if performed in a group format.

Rehabilitation research focusing on regaining lost physical or cognitive functions were highly requested (85%), followed by research on interventions focusing on learning strategies to manage tasks and activities that can be difficult to perform after stroke (59%). Interventions focusing on finding new/alternative ways of performing daily activities (including provision of assistive devices/housing adaptations) were of interest for a good third (38%).

One theme that emerged from the open-ended responses was the importance of taking a broad spectrum of individual needs into account to promote participation in rehabilitation research. It was related to the foci of rehabilitation interventions of interest such as *“everyday life”* and *“psychological stress”.* A recommendation regarding the form of the rehabilitation interventions was to offer interventions to similar target groups that shared similar impairments or problems: *“Coordination can be done with other neurological diseases, for example, Parkinson’s”.* The participants also stated a need of adapting rehabilitation to participants with different types and levels of impairments, bearing in mind their age and life circumstances: *“An important thing is to see the difference in age. What I, a woman not even 40 with small kids, need compared to somebody newly retired or in an age of 80 needs when it comes to rehabilitation should differ in some respects. Not the least cognitively. … Most people who get strokes are old and this causes trouble for us younger. This must be taken into consideration, so we can get help as well”.* Furthermore, some participants highlighted individual incentives that could encourage participation in rehabilitation research. They indicated that those who have actual needs of rehabilitation may be more positive than others.

Another theme focused on personal characteristics of participants and those who provided the interventions that would promote participation in rehabilitation research. Genuine interest and motivation among the potential participants as well as confidence and ability to communicate were mentioned: *“That they have an “open mind” and want to improve their situation although the years have past”* and *“That they can and dare to formulate their thoughts”.* Lastly, they stressed that the approach and competence of those who provided the intervention are essential, reflected by statements like *“Kindness and patience”* and *“Do not hurry”*.

## Discussion

In this study, we embraced a user perspective and explored opportunities to recruit stroke survivors as participants in rehabilitation research, which is an urgent methodological challenge for many researchers [2]. This study contributed new knowledge that could develop methodological awareness and strategies related to recruitment of study participants. The overall results show that there are stroke survivors in Sweden who are interested in participating in rehabilitation research, especially younger persons and those who experienced a stroke more recently, whereas older persons are more hesitant. This is partly in line with earlier research that revealed older age to be a challenging factor during recruitment for stroke research [8]. The result regarding preferred administration modes indicates that digitized or phone administration do not guarantee high response rates to surveys targeting stroke survivors.

The answers to the open-ended question stress that it is important that the content of rehabilitation research consider a broad spectrum of needs, not only type of disability, but also age and life situation. For instance, some of the younger participants in our sample stressed that their needs were not taken into account because they were put together with older persons who had other expectations and needs. This may reflect how local rehabilitation services in general are organized, and carried out. For example, where persons with the same diagnosis risk receiving the same rehabilitation without consideration for individual needs based on person-centered care [24]. Furthermore, as the composition of groups seems to be a key factor to achieve positive rehabilitation results, one solution might be to coordinate groups based on similar experiences rather than diagnosis, as suggested by participants in this study.

Although earlier research has documented that there are several challenging factors regarding recruitment to stroke research [7–9, 11], an encouraging result in our study is that among our participants, there is a strong interest for participating in rehabilitation research. Still, there might be a gap between being interested and actually attending. One reason might be the format of the rehabilitation services that are provided. Our results show that willingness to contribute to rehabilitation research is a common reason to participate – reflecting previous research in other populations [25], and that meeting others in the same situation is a strong incentive. This may reflect the high interest for group-based interventions, also confirming recent research where benefits of such approaches in rehabilitation research targeting stroke survivors have been revealed [26]. However, one should bare in mind that our sample is biased in favour of research, and we do not know the viewpoint of those who chose not to participate in the study.

Not only is the format of rehabilitation interventions important, but also their foci. Our study highlights that many stroke survivors prioritize rehabilitation research focusing on regaining lost physical or cognitive functions. This is presumably associated with a strong hope for recovery [27] and not surprising. Similar results were reported in another recent Swedish study with stroke survivors [28], showing the most prioritized areas of research to be ‘balance and walking difficulties’ as well as ‘post-stroke fatigue’. However, that study mainly targeted areas of stroke impairment whereas our study included questions also focusing on compensatory and lifestyle rehabilitation interventions. The responses to the open-ended question in our study highlight the importance of including individuals who had a long term post stroke experience. Even so, longer time since stroke onset seems to be related to a decreased interest to participate in rehabilitation research. This might be explained by the individual adaptation to the changed life situation following stroke, similar to the findings reported by Norlander et al. [29] who found that those who had lived many years since they first fell ill with stroke had adapted to the remaining consequences into “a new normal me”. Thus, rehabilitation interventions as well as rehabilitation research that aim to support this adaptation process should be offered when a person who had a stroke is struggling to manage everyday life and try to re-integrate in the community despite remaining impairments.

A specific result worth emphasizing is that a rather high proportion of our participants mentioned that obtaining support to travel to where the rehabilitation takes place is a key issue. Many stroke survivors cease to drive a car [30] and find it difficult to travel with public transport due to physical and cognitive impairments [31]. Our previous research has elucidated the importance of accessible transport for stroke survivors with cognitive impairments [32]. Although many countries provide Special Transport Services (STS) for people with disabilities, such services are very regulated [33]. Thus, there is an urge for society to provide accessible public transport. Another issue regarding transport is the cost associated with getting to the rehabilitation setting [34]. Compensating participants for such costs might result in a more successful recruitment, but although offering free journeys might be a good solution, it implies higher project expenses that should be covered by funding schemes and proposals.

The importance of the competence of the investigators and those who provide the intervention in rehabilitation research cannot be overestimated, especially in terms of their skills. Carlstedt et al. [26] asserted that the need of the individuals who participated in rehabilitation research must be considered and adapted accordingly. Sufficient skills training and practice of researchers and practitioners engaged in rehabilitation research should therefore be prioritized [8, 9].

Moreover, to promote study participation in rehabilitation research, aspects such as participant interest and motivation are important. Evaluations of readiness for making behavioral changes according to Prochaska’s and DiClemente’s change model [35] might be useful during the recruitment process. Such evaluations could also be used to individualize rehabilitation programs not only on participants’ needs but also based on their different motivation levels.

One limitation and weakness of the present study is the unknown overall response rate, which as such is related to the study rationale. As this study was initiated based on the experiences from a previous study, it ended up with a very low response rate despite major efforts to recruit a reasonable sample (26). Therefore, the present study relied on a sampling procedure striving to utilize several complementary strategies, and the weaknesses are a direct consequence of the recruitment procedure. Moreover, the included subjects are likely biased towards being stroke survivors willing to participate in rehabilitation research. Despite major efforts, we were unable to achieve a high response rate and recruit a larger sample. This is reflective of the challenges in obtaining responses to surveys [1], emphasizing the importance of the study as such. Still, the knowledge and experiences gained from the present study are important for rehabilitation researchers and could be used to develop more efficient recruitment strategies.

Furthermore, the composition of the study sample should be mentioned. The prevalence of cognitive impairments in our study was low compared to in the general population of stroke survivors [36]. On the other hand, the proportion of possible depression was twice as high as compared to a recent review including stroke survivors up to 5 years after stroke [37]. This implies that our results are not representative for the stroke survivor population. Reflecting on the high proportion of possible depression in our sample, especially in the light of the results of Boxall et al. [8] who showed that depression is a challenging factor for recruiting stroke survivors to clinical trials, we could have expected a lower interest to participate in rehabilitation research in our study. However, we did not find any differences regarding the interest to participate in rehabilitation research between those with possible depression and those without.

A strength of the present study is that we collected data from potential research participants’ perspective, and not from professionals. We utilized several recruitment and administration modes, and had generous inclusion criteria to reach a broad sample of stroke survivors. We used quantitative and qualitative data collection, making it possible to answer the research questions from different perspectives in a complementary manner. The finding that the vast majority of the responding stroke survivors prefer to be contacted by postal letter and use paper forms to respond speaks for consciousness in this regard when using surveys with this specific population. In an international perspective, it is important to keep in mind that Sweden is among the most digitalized countries in the world [38] and increasing proportions of older people and people with disabilities use the Internet and digital technologies [39]. Our findings do not imply that stroke survivors are negative towards using new modes of communication. For instance, previous research shows that people post-stroke who use digital modes of communication are positive towards using apps as a health-monitoring tool [40], something that has been further developed during the covid-pandemic. Still, the fact is that 15% of the population in Sweden aged 65+ do not use internet at all [37], and the majority of stroke survivors belong to this age group.

## Conclusions

Although recruitment of stroke survivors to rehabilitation research is a challenging task, there are study participants who are highly interested. Recommending potentially successful methodological strategies based on the results of the present study, a range of personal and external aspects should be taken into account to achieve high participation rates in rehabilitation research targeting stroke survivors. The importance of addressing needs and prerequisites in an individualized manner should not be underestimated and might be a useful strategy to recruitment success. Although the study sample was small and presumably biased towards stroke survivors who already had an interest to participate in rehabilitation research, the results can be used to guide researchers when designing and planning for new projects. A noteworthy methodological implication is that although we offered several administrative modes, the majority used the traditional paper form and wanted to be contacted through postal mail. This is contradictory to the ongoing digitalization of society and needs to be further explored.

## Data Availability

The data used in this study contains sensitive information about the study participants and they did not provide consent for public data sharing. The current approval by the Regional Ethical Review Board in Lund, Sweden (see above) does not include data sharing. A minimal data set could be shared by request from a qualified academic investigator for the sole purpose of replicating the present study, provided the data transfer is in agreement with EU legislation on the general data protection regulation and approval by the Swedish Ethical Review Authority. Contact information: Department of Health Sciences, Lund University Box 157, 221 00 Lund, Sweden DHSdataaccess@med.lu.se. Principal investigator: Susanne.Iwarsson@med.lu.se

## Abbreviations

GDS-20: Geriatric Depression Scale
LSR: Lund Stroke Register
STS: Special Transport Services
SIS: Stroke Impact Scale 2.0
GSE: The General self-efficacy scale
